# A Retrospective Analysis of Thyroid Storm at a Tertiary Care Center in Northern India

**DOI:** 10.7759/cureus.99537

**Published:** 2025-12-18

**Authors:** Manisha Mallavarapu, Ritesh Kumar, Soumya Ranjan Mohanty, Reshma Manayankath, Neeraj Kumar Agrawal

**Affiliations:** 1 Department of Endocrinology and Metabolism, Institute of Medical Sciences, Banaras Hindu University, Varanasi, IND

**Keywords:** burch-wartofsky score, impending thyroid storm, thyroid storm, thyrotoxic crisis, thyrotoxic storm

## Abstract

Introduction and aim: Thyroid storm is a rare, yet life‑threatening form of thyrotoxicosis, featured by exaggerated clinical symptoms and signs along with multiorgan dysfunction. It is almost fatal (80-100%) if left untreated, and even with treatment, it carries a mortality rate of 50%. This study aimed to investigate the demographic profiles, clinical and biochemical features, and outcomes of patients hospitalized with thyroid storm at our institution.

Material and methods: This retrospective cohort study was conducted in the department of endocrinology and metabolism from January 2015 to July 2024. Medical records of patients diagnosed and admitted with thyroid storm or impending storm and those with calculated Burch-Wartofsky score (BWS) of ≥45 were reviewed. Data on demographics, clinical features, laboratory results, treatment, and outcomes were collected from hospital records. Incomplete records and cases with BWS <45 were excluded. Data were entered into Microsoft Excel and analyzed using R software (Vienna, Austria: R Foundation for Statistical Computing) with descriptive statistics applied.

Results: A total of 15 patients were included in the study. Of these, 73% presented with thyroid storm, while 26% presented with impending storm. Ninety-two percent of the patients were female, and the mean age was 46.07±12.62 years. Graves’ disease was the underlying cause in all patients. Twenty-one percent had newly detected thyrotoxicosis, whereas 79% were known cases of Graves’ disease. Cardiovascular dysfunction was observed in 92% of patients, with atrial fibrillation seen in 28%. This was followed by gastrointestinal dysfunction in 85% and central nervous system dysfunction in 42% of patients, most commonly presenting as mild agitation. A precipitating factor was identified in 57% of cases, with respiratory tract infection being the most common. Deranged liver function tests were noted in 42% of patients. Among the 11 patients with thyrotoxic storm, one patient expired within 72 h of admission due to heart failure.

Conclusion: In our study, Graves’ disease was the underlying cause of thyrotoxicosis storm in all the patients. A predominant cardiovascular dysfunction, followed by gastrointestinal dysfunction, was the most common presentation. Notably, central nervous system (CNS) dysfunction was observed less frequently and with mild severity than reported in other studies. Thyroid storm is a potentially lethal presentation of thyrotoxicosis; early diagnosis and prompt treatment are lifesaving.

## Introduction

Thyroid storm (TS), also known as thyrotoxic crisis, is a rare but life-threatening complication of thyrotoxicosis, characterized by a rapid deterioration of physiological functions. The “crisis of exophthalmic goiter” was the first description given to these patients, noted among too critically ill patients undergoing surgery, in 1928 by Lahey [1]. It is a multisystem condition, with systemic decompensation, associated with severe, acute manifestations of hyperthyroidism that include fever, tachycardia, delirium, gastrointestinal symptoms, and multiorgan dysfunction. Thyrotoxic crisis can be due to various etiologies of hyperthyroidism, but Graves’ disease, toxic nodule, and multinodular goiter are the common causes. These conditions may lead to thyroid storm in the presence of some precipitating factors, such as surgery, infections, trauma, iodine exposure [2-7].

Thyroid storm is a medical emergency condition; it needs immediate recognition and prompt intervention to reduce its associated morbidity and mortality. The estimated mortality rate for thyroid storm ranges from 8% to 25%, with multiorgan dysfunction being the leading reported cause of death, followed by congestive heart failure and respiratory failure [8]. The current guidelines from the American Thyroid Association (ATA) advocate for an aggressive treatment approach that includes inhibiting thyroid hormone synthesis and secretion, blocking peripheral thyroid hormone effects, addressing the underlying precipitating factor, and managing systemic decompensation [9]. Despite advances in medical care, thyroid storm remains a significant cause of morbidity and mortality due to delayed diagnosis and inadequate or suboptimal treatment.

In 1993, Burch and Wartofsky introduced a quantitative diagnostic tool that is now considered to be a reliable criterion for detecting TS [2]. This method assigns points for dysfunction of the thermoregulatory, central nervous, gastrointestinal (GI)-hepatic, and cardiovascular systems, with increasing points given for greater severity of dysfunction. A total score of 45 or higher is considered highly sensitive and suggestive of TS, and this threshold has been extensively accepted in the literature [2]. The Japan Thyroid Association (JTA) diagnostic criteria for thyroid storm are also an accepted score in diagnosing thyroid storm, but a prerequisite of thyroid function tests is needed [8]. Furthermore, this criterion is not quantitative; it considers only the presence of a defined clinical feature.

The rarity of thyroid storm poses challenges in clinical practice, as the diagnosis is frequently delayed or initially missed due to its overlap with other acute medical conditions. Early recognition is vital; however, variability in clinical presentation and limited clinician familiarity often lead to underdiagnosis. In this context, retrospective analyses of thyroid storm cases are invaluable, as they provide deeper insights into their clinical presentations, precipitating factors, and organ-system involvement. These insights can help refine diagnostic practices, improve risk stratification, and shape management strategies that lower mortality and support better patient outcomes.

## Materials and methods

This study retrospectively reviews the clinical presentation, etiology, and diagnostic criteria of thyroid storm, and investigates factors contributing to mortality and adverse outcomes. This retrospective study was conducted at the Department of Endocrinology and Metabolism, Institute of Medical Sciences, Banaras Hindu University (BHU), Varanasi, Uttar Pradesh, India. Data were collected on all patients admitted between January 2015 and July 2024 with a diagnosis of thyroid storm (TS) or impending thyroid storm. Among them, those with Burch-Wartofsky score (BWS) of 45 or more were included in the study. In cases where the BWS was not explicitly mentioned in the medical records, it was calculated based on the available clinical and laboratory data. Patients with incomplete or missing medical records, or when the BWS score is less than 45, were excluded.

Data were collected from hospital medical records. The following parameters were obtained from the records: demographic details, including age, gender, and comorbidities; clinical presentation and triggering factors; laboratory results, including thyroid function tests and other relevant investigations (e.g., liver and renal function); treatment given; and outcomes, including mortality, ICU admission, recurrence, and long-term follow-up data. No informed consent was obtained, as this is a retrospective study that collects data from hospital records.

Thyroid-stimulating hormone (TSH) and total thyroxine (T4) levels were measured using the chemiluminescence immunoassay (CLIA) method with UniCel DxI 800 (Brea, CA: Beckman Coulter, Inc.). The manufacturer’s specified normal reference range for TSH is 0.4-5.3 µIU/mL and for T4 is 4.8-11.72 µg/dL. TSH receptor antibody was assayed by ELISA using ElisaRSR TRAb Fast (Cardiff, UK: RSR Limited). Data were collected and entered into Microsoft Excel and analyzed using R software version 4.4.1 (Vienna, Austria: R Foundation for Statistical Computing). Descriptive statistics were used to summarize demographic, clinical, and laboratory data. Frequencies and percentages were reported for categorical variables, whereas means and standard deviations (SDs) were reported for continuous variables.

## Results

A total of 15 patients had been admitted to our endocrinology department with thyrotoxic crisis over the duration of 10 years. Out of them, four patients (26.6%) had a BWS score between 25 and 44, suggestive of impending storm, and 11 patients (73.3%) had a BWS score of 45 or more, suggesting thyroid storm. Data from these 11 patients had been further analyzed.

**Figure 1:**
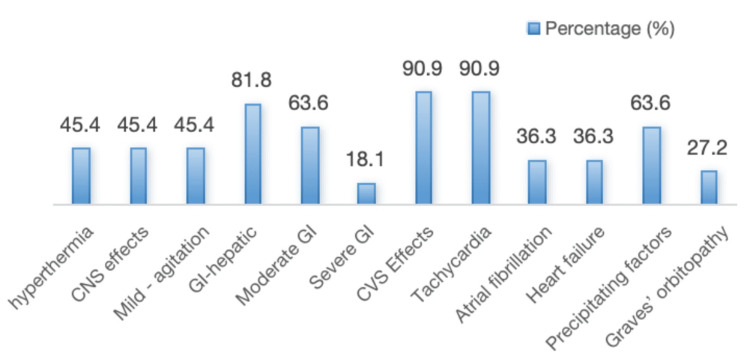
Clinical features among thyroid storm patients. GI: gastrointestinal manifestations; CVS: cardiovascular system

The baseline and clinical characteristics of these patients have been presented in Table 1. Among the patients with thyroid storm, the mean age was 47.9±6.8 years, with female predominance of 10 out of 11 subjects (90.9%). The mean BWS score was 51.8±6.6. However, only eight out of 11 patients were diagnosed with definite thyroid storm by the Japan Thyroid Association (JTA) criteria. All our patients had Graves’ disease as the only etiology of thyrotoxicosis. Graves’ orbitopathy was present in three subjects (27.2%). The median duration of Graves’ disease was 22 months (ranging from two months to 120 months). Three patients presented with thyroid storm as their first presentation of thyrotoxicosis (27.3%), while eight patients were known cases of thyrotoxicosis (72.7%). Regarding the clinical features of thyroid storm at presentation, the most common system involved was cardiovascular (90.9%), followed by the gastrointestinal system (81.8%) (Figure 1). Among patients with cardiovascular dysfunction, almost all had severe tachycardia, i.e., heart rate ≥140 bpm, while atrial fibrillation was seen in four (36.3%) patients, and congestive heart failure was seen in four (36.3%) patients. Gastrointestinal dysfunction was seen in nine (81.8%) patients, with moderate involvement (nausea, diarrhea, abdominal pain) in seven (63.6%) patients, while severe manifestation (unexplained jaundice) was seen in two (18.1%) patients. While central nervous system dysfunction was seen in less than half of the patients (45.4%), i.e., five out of all, almost all had only mild agitation. None of them had delirium, psychosis, seizure, or coma. Hyperthermia was seen in only five of them (45.4%), mostly a mild to moderate rise in temperature. None had severe hyperthermia (>104°F).

**Table 1:** Clinical, biochemical, and treatment outcomes among patients with thyroid storm. JTA: Japan Thyroid Association; ANC: absolute neutrophil count; AST: aspartate aminotransferase; ALT: alanine aminotransferase; ALP: alkaline phosphatase; Na: sodium; K: potassium; TSH: thyroid-stimulating hormone; T4: thyroxine; IQR: interquartile range

Clinical features and treatment response	Actual values
Mean age in years (SD)	47.9±6.8
Female gender, n (%)	10 (90.9%)
Newly diagnosed thyrotoxicosis, n (%)	3 (27.3%)
Known case of thyrotoxicosis, n (%)	8 (72.7%)
Median duration of thyrotoxicosis (IQR in months)	22 (20-84)
Recurrent thyroid storm, n (%)	1 (9%)
Graves’ disease as etiology (%)	100%
Mean BWS score	51.8±6.6
Definite thyroid storm (TS1) by JTA criteria, n (%)	8 (72.7%)
First combination, n (%)	5 (45.4%)
Second combination, n (%)	3 (27.2%)
Mortality, n (%)	1 (9%)
Laboratory investigations	
Investigation (normal range)	Mean±SD
Hemoglobin (13-17 g/dL)	11.3±0.8
Total WBC count (4,000-10,000 cells/mm^3^)	8500±2210
ANC (2,000-7,000 cells/mm^3^)	5621±3032
Platelet count (1.5-4.5 × 10⁵/mm^3^)	1.8±0.9
Total bilirubin (0.3-1.2 mg/dL)	1.2±0.6
AST/ALT (<40/<34 U/L)	47 (27-66)/34 (21-48)
ALP (<240 U/L)	172±63.3
Na (135-145 mmol/L)	138.7±4.8
K (3.5-5.3 mmol/L)	3.5±0.39
Total T4 (4.87-11.72 µg/dL)	21.44±2.91
TSH (0.35-4.94 µIU/mL)	<0.01

**Figure 2:**
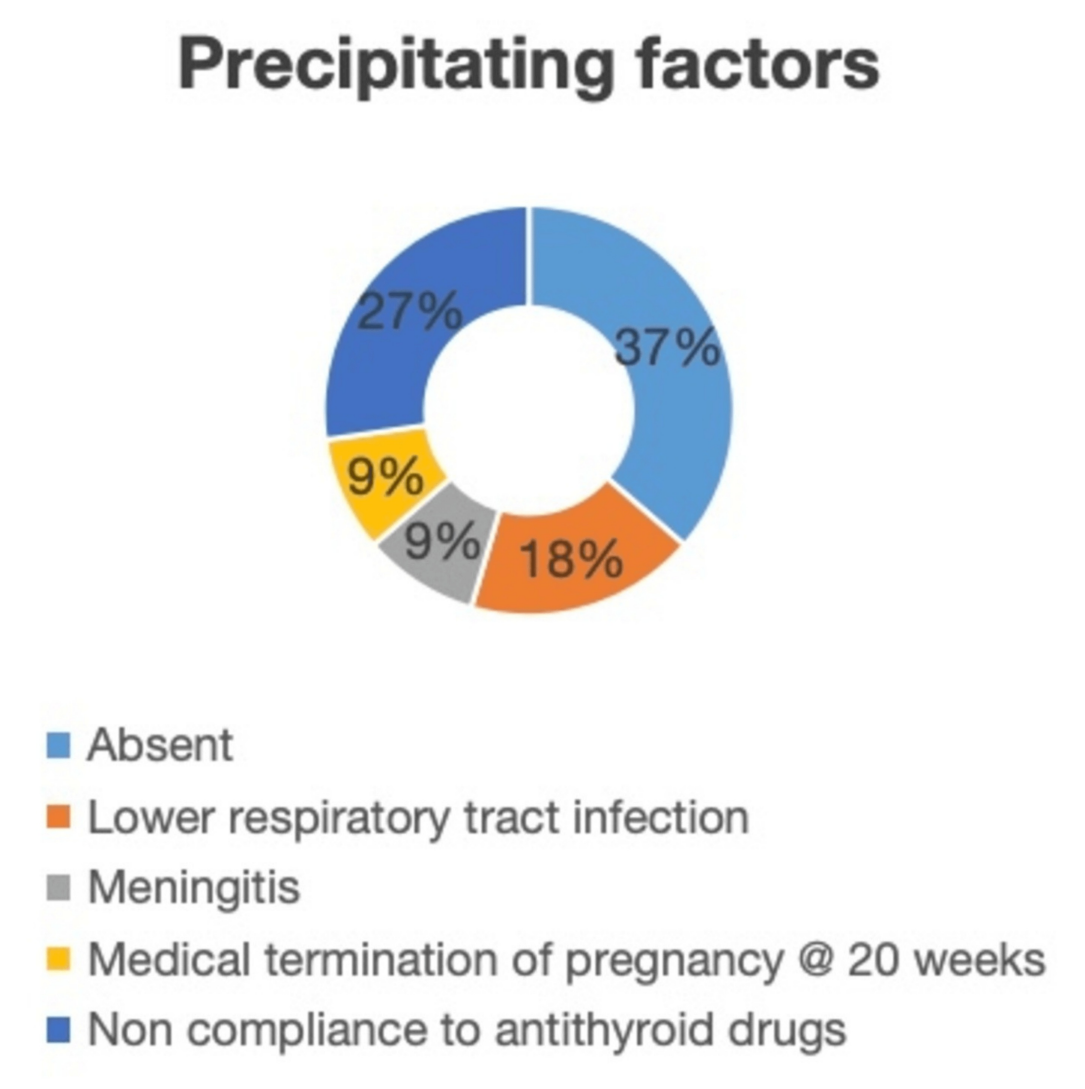
Precipitating factors among thyroid storm patients.

A precipitating event/factor was noted in more than half of patients (63.7%) (Figure 2). Out of seven patients with a precipitating event, three patients were non-compliant to antithyroid drugs, two patients had lower respiratory tract infection, one had meningitis, and one patient developed a storm after medical termination of pregnancy at 20 weeks (due to fetal anencephaly on anomaly scan). No precipitating factor was noted in four patients.

On laboratory investigation, mean hemoglobin levels were 11.3±0.8 g/dL, WBC count 8500±2210 cells/mm^3^, and absolute neutrophil count 5621±3032 cells/mm^3^. The median of total bilirubin, SGOT, and SGPT were within normal limits, while ALP levels were slightly elevated above the upper limit of normal. Only two patients had hypokalemia; otherwise, serum electrolytes were normal in the rest. Mean total T4 level was 21.44±2.91 µg/dL, whereas TSH was <0.01 µIU/mL in all but one patient, who had a TSH level of 0.02 µIU/mL.

With regard to the management of thyroid storm in our patients, beta-blockers, especially propranolol, were used in nine patients (81.8%), whereas a calcium channel blocker was used in one patient (9%). Among antithyroid drugs, carbimazole was used in six (54.5%) patients, propylthiouracil in three (27.2%), Lugol's iodine 2% in four (36.3%), lithium in two (18.1%), and cholestyramine in three (27.2%) patients. Glucocorticoids were given to all patients, with hydrocortisone to seven (63.6%) patients and dexamethasone to four (36.3%) patients. None of our patients underwent plasmapheresis. Out of 11 patients, one patient needed ICU admission, and the same patient expired within 48 h of admission due to severe congestive heart failure. In our study, the mortality rate was 9%. Long-term follow-up data were lacking. On tracing, six patients' long-term data were available; among them, two had achieved remission with carbimazole therapy, two were on low-dose carbimazole therapy, and two patients underwent successful radioiodine ablation. One patient developed recurrent thyroid storm three months after the first episode due to non-compliance with antithyroid drugs.

## Discussion

Thyroid storm (TS) is an uncommon condition, but a critical manifestation of thyrotoxicosis, characterized by an acute and severe exacerbation of clinical symptoms. It remains a potentially life-threatening emergency, necessitating prompt recognition and aggressive management. Our study highlights the multifaceted nature of thyroid storm, with both clinical and laboratory findings supporting its significant morbidity and mortality.

In our cohort of 15 patients, 11 met the diagnostic criteria for thyroid storm, and most were middle-aged women (mean age: 47.9±6.8 years), reflecting the well-established gender predominance seen in thyroid storm [10-12]. The female preponderance in thyroid storm has been consistently reported, likely due to the higher incidence of Graves' disease, the most common etiology of thyrotoxicosis in women. Notably, three patients (27.3%) presented with thyroid storm as their first manifestation of thyrotoxicosis, highlighting the potential for thyroid storm to occur in patients without prior history of hyperthyroidism. In some studies, up to 40% of patients presented with thyroid storm were newly diagnosed cases of thyrotoxicosis [10,13]. This underlines the importance of considering thyroid storm in the differential diagnosis of acute, unexplained systemic illness.

This study confirms Graves' disease as a primary etiology of thyrotoxicosis, consistent with existing literature that identifies it as a leading cause of thyroid storm [10,11,13]. The clinical presentation of thyroid storm in our study was dominated by cardiovascular dysfunction, with tachycardia (heart rate ≥140 bpm) seen in nearly all patients, and atrial fibrillation and congestive heart failure in 36.3% each. These findings underscore the cardiovascular complications commonly associated with thyroid storm, where the excessive thyroid hormones increase heart rate and contractility, often leading to arrhythmias and heart failure [14]. As in previous studies, severe tachycardia was the most prominent cardiovascular sign in our cohort [10-13]. It highlights the need for immediate control of heart rate to stabilize patients and prevent further complications. While our study did not find severe neurological symptoms such as delirium or seizures, mild agitation was observed in nearly half of the patients (45.4%), which is also a common feature of thyroid storm, reflecting its impact on the central nervous system [13]. This signifies the predominant cardiovascular involvement over neurological involvement in our study. These findings were consistent with the other Indian study by Lath et al. [10]. However, few studies have reported more pronounced neurological involvement in thyroid storm [8,11,13].

Gastrointestinal dysfunction was present in 81.8% of patients, with diarrhea and abdominal pain being the most common. In two patients, severe gastrointestinal manifestations like unexplained jaundice were noted, which may reflect more severe hepatic involvement. Thyroid storm can lead to elevated liver enzymes and even liver failure in extreme cases, although liver function tests were normal in most of our patients. Alkaline phosphatase (ALP) was elevated in some patients (27.2%), which may reflect hepatic congestion or cholestasis, a finding also reported in other studies [15]. This is consistent with prior literature, which notes that liver dysfunction can occur in up to 20% of thyroid storm cases, though it is often reversible with treatment [11]. Importantly, electrolyte abnormalities were minimal, with only two patients showing hypokalemia. Despite these widespread systemic effects, hyperthermia was observed in only 45.4% of patients, a lower incidence than in other studies in which fever is a hallmark of thyroid storm [10]. The fever typically results from metabolic acceleration caused by excessive thyroid hormone levels, although its absence does not exclude the diagnosis of thyroid storm. The presence of hyperthermia in only a few patients indicates the heterogeneous nature of thyroid storm, in which symptoms may vary widely in intensity.

In our study, 63.7% of patients had identifiable precipitating factors, such as infection (lower respiratory tract infection), non-compliance with antithyroid medications, and even medical termination of pregnancy. This highlights the importance of recognizing the factors that can trigger thyroid storm in patients with underlying thyrotoxicosis. In particular, infection has been well documented as a major precipitating event, as it induces an inflammatory response that exacerbates thyroid hormone release [16]. Also, these precipitating factors release more thyroid hormones from their binding sites or increase the sensitivity of receptors in tissues. The increased receptor sensitivity could be due to a rise in adrenergic receptor density on target cells or through changes in signaling pathways after receptor activation [17]. Non-compliance with antithyroid drugs was noted in three patients, underscoring the role of patient adherence in preventing thyroid storm [13]. It also points to the need for better patient education regarding the potential dangers of untreated or inadequately treated hyperthyroidism. Our cohort’s laboratory results revealed a mean total T4 level of 21.44±2.91 µg/dL, which was significantly higher than the normal reference range. But there doesn't seem to be a clear connection between thyroid hormone levels and the characteristics of thyroid storm. Studies have shown that both groups, patients either experiencing thyroid storm or those with only overt thyrotoxicosis, have similar levels of free thyroid hormones [8]. Its occurrence is also highly unpredictable. Despite well-defined diagnostic criteria and treatment approaches, the exact pathogenesis of thyroid storm remains incompletely understood [18].

The management of thyroid storm in our cohort primarily involved beta-blockers, such as propranolol, as they are essential for controlling tachycardia and preventing life-threatening arrhythmias. Antithyroid drugs, including carbimazole and propylthiouracil, help block the synthesis of thyroid hormones. In a few cases, Lugol's iodine and lithium were used instead of thionamides due to coexisting liver dysfunction at presentation. Glucocorticoids, primarily hydrocortisone, were administered to reduce peripheral conversion of T4 to T3 and help mitigate inflammation. These treatment strategies align with current guidelines that emphasize beta-blockers, antithyroid agents, and glucocorticoids as the cornerstone of therapy [9]. The mortality rate of 9% in our study is lower than the 20-30% mortality rates reported in some other cohorts, reflecting the effectiveness of early diagnosis and aggressive management. Only one patient in our study died due to severe congestive heart failure, highlighting the potential for rapid deterioration despite appropriate treatment. According to a study by Ono et al., the most common cause of mortality was cardiovascular dysfunction [12].

This study has a few limitations, including its retrospective design and the relatively small sample size, which may limit the generalizability of the findings. Additionally, the absence of a control group prevents us from directly comparing the outcomes with those of patients without thyroid storm. Future studies with larger multicenter cohorts would help validate these findings and provide clearer insights into optimal management strategies for thyroid storm.

## Conclusions

Thyroid storm remains a rare but potentially fatal complication of thyrotoxicosis. Early recognition, prompt diagnosis, and aggressive treatment are essential for improving outcomes. In our cohort, cardiovascular manifestations were predominantly seen, followed by gastrointestinal symptoms, while neurological features were less commonly observed. Our study highlights the critical role of cardiovascular support, antithyroid medications, and glucocorticoids in managing thyroid storm and underscores the importance of identifying precipitating factors and ensuring long-term patient adherence to prevent recurrence.
